# Burden of Alcoholic Liver Disease in a Tertiary Care Center: A Descriptive Cross-sectional Study

**DOI:** 10.31729/jnma.4631

**Published:** 2019-10-31

**Authors:** Shikha Rizal, Bishal Raj Joshi, Arambam Giridhari Singh

**Affiliations:** 1 Department of Biochemistry, Nobel Medical College and Teaching Hospital, Biratnagar, Nepal

**Keywords:** *alanine transaminase*, *alcoholic liver disease*, *aspartate transaminase*, *de-ritis ratio*, *gamma glutamyltransferase*

## Abstract

**Introduction:**

Alcoholic liver disease is a serious health problem related to an unhealthy lifestyle. The three most widely recognized forms of alcoholic liver disease are alcoholic fatty liver, acute alcoholic hepatitis, and alcoholic cirrhosis. The main aim of our study is to find out the prevalence of alcoholic liver disease in tertiary care center.

**Methods:**

A descriptive cross-sectional study was conducted among inpatient cases admitted in the medicine department of tertiary care center from 1^st^ June 2018 to 31^st^ May 2019. Ethical approval was taken for the study. Convenience sampling method was used. All the biochemical parameters were expressed as mean±standard deviation for each group and point estimate at 95% Confidence Interval was calculated along with frequency and proportion for binary data.

**Results:**

Prevalence of alcoholic liver disease is 50 (50%) at a 95% Confidence Interval (40.2%-59.8%) and non-alcoholic fatty liver disease is also the same. The mean age of alcoholic liver disease was 59±12 years where as the mean age for non-alcoholic fatty liver disease was 46±18 years. Out of fifty patients of alcoholic liver disease, majority 48 (96%) of the cases were males which suggests that the prevalence of alcoholic liver disease is very common in males. Similarly, for non-alcoholic fatty liver disease, prevalence was 34 (68%) showing higher prevalence than that of females.

**Conclusions:**

Prevalence of alcoholic liver disease is low compared to previous studies done in the similar settings. Monitoring these biochemical parameters in alcoholic liver disease at early stage could guide in planning the protocol for the initial treatment.

## INTRODUCTION

Fatty Liver Disease (FLD) is a common disease which can be subdivided according to its cause into: alcoholic fatty liver disease, a type of alcoholic liver disease (ALD) and non-alcoholic fatty liver disease (NAFLD).^1^ ALD is a general term used to refer to a spectrum of alcohol-related liver injuries including fatty liver, alcoholic hepatitis and alcoholic cirrhosis,^2^ whereas NAFLD is a spectrum of liver diseases characterized by the presence of ectopic fat in the liver, which cannot be explained by alcohol consumption resulting in simple steatosis to non-alcoholic steatohepatitis (NASH) and cirrhosis.^3^

Alcohol is a well established hepatotoxin whose consumption is always associated with risk of development of ALD, with no absolute threshold of alcohol consumption necessary for the development of liver injury.^4^ About 50% of liver cirrhosis are alcohol induced and 10-20% of alcoholics will suffer liver cirrhosis.^5^ The prevalence of alcohol consumption among Nepalese population was found to be 67%, but the degree of liver injury due to alcoholism in Nepalese community is still unknown.^6^

The main aim of this study is to find out the prevalence of alcoholic liver disease in inpatients of medicine department of Nobel Medical College and Teaching Hospital.

## METHODS

A descriptive cross-sectional study was conducted on a total of 100 subjects who were admitted in the medicine department of Nobel Medical College and Teaching Hospital, Biratnagar. The study was conducted from 1^st^ June, 2018 to 31^st^ May, 2019. Ethical approval was taken from the Institutional Review Committee of Nobel Medical College and Teaching Hospital. Informed consent was taken from each patient before data collection. Inclusion criteria for participation in our study is admission to in medicine department of Nobel Medical College and Teaching Hospital with liver disease and age >20 years. Exclusion criteria for participation in our study was less than 20 years of age. Convenience sampling was used to collect data. Sample size was determined by using following formula:

n = Z^2^ × (p × q)/e^2^

   = 92

where,

n = sample size
Z = 1.96 at 95% Cl
p = prevalence= 60% (educated guess)
q = 1-p
e = margin of error= 10%

Taking non-response rate of 8%, total sample size was taken as 100.

Diagnosis of ALD was made on the basis of history of alcohol intake, clinical examination and ultrasonography of the abdomen. Similarly, diagnosis of NAFLD was made based on the presence of metabolic syndrome, clinical examination and ultrasonography of the abdomen. Blood samples from all the patients were collected from antecubital vein after an overnight fast. Blood was allowed to clot and serum was separated. Serum from all patients were analyzed for the following biochemical parameters: aspartate Transaminase (AST), Alanine Transaminase (ALT), Alkaline Phosphatase (ALP) and Gamma Glutamyl Transferase (GGT) using VITREOUS auto analyzer in the central laboratory of NoMCTH. De-ritis ratio was calculated by taking the ratio between AST/ALT.

The data were collected and entered in MS-Excel and analyzed using the Statistical Package for Social Sciences (SPSS) version 16 software. Point estimate at 95% confidence interval was calculated along with frequency and proportion for binary data.

## RESULTS

Prevalence of alcoholic liver disease is 50 (50%) at 95% of C.I (40-60) and non-alcoholic liver disease is also the same. The mean age of ALD was 59±12 years where as the mean age for NAFLD was 46±18 years. Out of fifty patients of ALD, majority 48 (96%) of the cases were males which suggests that the prevalence of ALD is very common in males. Similarly for NAFLD the prevalence was 34 (68%) showing higher prevalence than that of females.

**Table 1:** Distribution of disease according to age and sex.

Subjects	Age in years (n=50)	Males	Females	Total
	Mean±SD	n (%)	n (%)	n (%)
Alcoholic liver disease	59±12	48 (96)	2 (4)	50 (100)
Non - alcoholic fatty liver disease	46±18	34 (68)	16 (32)	50 (100)

There is a marked increase in serum AST levels in cases of AL (Table 2).

**Table 2:** Biochemical parameters in patients with ALD and NAFLD (n=100).

PARAMETERS		ALD (n=50)	NAFLD (n=50)
		Meant±SD	Mean±SD
AST (IU/L)	136.02±28.72	56.43±12.43
ALT (IU/L)	54.42±38.72	64.56±13.22
ALP (IU/L)	116.77±26.86	88.76±32.70
GGT (IU/L)	98.32±19.88	38.86±4.88

**Table 3:** Calculated De-ritis ratio in patients with ALD and NAFLD (n=100).

Ratio	ALD (n=50)	NAFLD (n=50)
	Mean±SD	Mean±SD
De-ritis ratio (AST/ALT)	2.49±0.8	0.87±0.27

De-ritis ratio more than 2 is seen in majority of the cases of ALD (Table 4).

**Table 4:** Distribution of De-ritis ratio in ALD and NAFLD.

	De-ritis ratio	ALD	NAFLD
	<2	8 (16)	50 (100)
	>2	42 (84)	0

## DISCUSSION

ALD and NAFLD are both serious health problems, both related to unhealthy lifestyle like excessive alcohol abuse and excessive food intake. Both ALD and NAFLD share a common clinical presentation, with very little knowledge regarding their prevalence in our population, resulting in serious health complication later.^11^

In our study, the mean age for ALD was 59±12 years, whereas the mean age for NAFLD was 46±18 years which was similar to other population based studies also.^12^ In our study, ALD was predominant in males which reflects that alcohol drinking is less common among Nepalese women. This was consistent with the study done in the USA showing male predominance with male:female ratio of 9:1.^13^ Similarly, male predominance was observed in NAFLD cases in our study which was consistent with the studies done in the USA, Japan and China.^13-15^

Similarly our study showed that the serum values of AST, ALP and GGT are higher in cases with ALD, the serum values of ALT was slightly higher in cases of NAFLD than in ALD.^5,16^ Rise in these liver enzyme indicate the leakage of hepatic intracellular enzyme into circulation indicating to be a marker for hepatocellular damage.^17^ Transaminase levels in ALD patients were AST dominant while those in NAFLD patients were ALT dominant.^5^ Our studies show a markedly higher value of GGT in cases with ALD. Similar results were seen in studies done by Atul, et al.^18^ which suggests that GGT can be a simple laboratory screening test for alcohol abuse, with quite good sensitivity but its specificity is poor.^18^ Elevated GGT level was related to biological effect of alcohol consumption rather than amount of alcohol consumed, which induces the protein expression and increased synthesis of GGT.^19^ Alcohol abuse causes damage to the liver cell causing release of GGT from microsomes into blood. Studies have shown that GGT has higher predictive value of ALD.^1,18,20^ Increase in serum levels of ALP along with GGT suggests a liver pathology.^19^

The De-ritis ratio was higher in patients with ALD (2.49±0.8) than that of NAFLD (0.87±0.27). Serum AST level was significantly higher than ALT in ALD groups which gave rise to higher De-ritis ratio, and this was similar to the studies done by Ram, et al.^21^ Junking, et al.^1^ Serum ALT is usually higher than AST in most causes of liver damage, but AST remains significantly higher in case of ALD. This might be because AST is a mitochondrial enzyme, acetaldehyde and free radicals produced during ethanol metabolism can lead to oxidative stress and lipid peroxidation causing mitochondrial injury which results in leakage of AST in the circulation as mAST is the more prevalent isoenzyme with approximately 80% of total AST activity in human liver contributed by mAST.^21^ Also there will be pyridoxal 5′-phosphate depletion in the liver of alcoholics and decreased hepatic ALT activity.^18^

According to findings in our study, majority 84% of the cases with ALD has De-ritis ratio greater than 2. This finding is consistent with many other studies done to evaluate De-ritis ratio in ALD and NAFLD.^18^ According to a study done by Nyblom and his associates, most patients with heavy alcohol drinking does not have AST:ALT ratio greater than 1 thus a high AST:ALT ratio (generally >2) suggests advanced alcoholic liver disease. The determination of the De-ritis ratio in alcoholic liver disease patients can thus be considered as a dependable marker, which has also been proved by international data.

## CONCLUSIONS

Prevalence of alcoholic liver disease is low compared to previous studies done in the similar settings. It is evident from the results of our study and the existing literature, that there are alterations in the biochemical parameters in both the cases. It is therefore concluded that these biochemical parameters can be used prior to invasive diagnostic procedure to support the clinical diagnosis and establish initial treatment plan. Monitoring these biochemical parameters in ALD at an early stage could guide in planning the protocol for the initial treatment.
